# Barriers to equitable healthcare services for under-five children in Ethiopia: a qualitative exploratory study

**DOI:** 10.1186/s12913-024-11074-0

**Published:** 2024-05-10

**Authors:** Hailu Fekadu, Wubegzier Mekonnen, Aynalem Adugna, Helmut Kloos, Damen Hailemariam

**Affiliations:** 1 Department of Public Health, Arsi University College of Health Science, Assela, Ethiopia; 2 School of Public Health, Addis Ababa University College of Health Science, Addis Ababa, Ethiopia; 3 Department of Geography, Planning and Environmental Sonoma State University, Sonoma, San Francisco, USA; 4 Department of Epidemiology and Biostatistics, University of California, San Francisco, USA

**Keywords:** Accessibility barriers, Inequity, Healthcare utilization, Under-five children, Ethiopia

## Abstract

**Background:**

Disparities in child healthcare service utilization are unacceptably high in Ethiopia. Nevertheless, little is known about underlying barriers to accessing child health services, especially among low socioeconomic subgroups and in remote areas. This study aims to identify barriers to equity in the use of child healthcare services in Ethiopia.

**Methods:**

Data were obtained from 20 key- informant interviews (KII) and 6 focus group discussions (FGD) with mothers and care givers. This study was conducted in Oromia Region, Arsi Zone, Zuway Dugda District from June 1–30, 2023. The study participants for this research were selected purposively. The information was collected based on the principle of saturation after sixteen consecutives interview were conducted. Both KII and FGD were audio-recorded and complementary notes were taken to record observations about the participants’ comments and their interactions. Each interview and FGD data were transcribed word-for-word in the local Afaan Oromo and Amaharic languages and then translated to English language. Finally, the data were analyzed thematically using NVivo 14 software and narrated in the linked pattern of child health service utilization.

**Results:**

This study identified six major themes which emerged as barriers to healthcare utilization equity for caregivers and their -under-five children. Barriers related to equity in low level of awareness regarding need, low socioeconomic status, geographical inaccessibility, barriers related to deficient healthcare system, community perception and cultural restrictions, and barriers of equity related to political instability and conflict*.* The most commonly recognized barriers of equity at the community level were political instability, conflict, and a tremendous distance to a health facility. Transportation challenges, poor functional services, closure of the health facility in working hours, and lack of proper planning to address the marginalized populations were identified barriers of equity at organizational or policy level.

**Conclusion:**

This study showed that inequity in child healthcare utilization is an important challenge confronting Ethiopia. To achieve equity, policy makers and planners need to change health policy and structure to be pro-poor. It is also necessary to improve the healthcare system to increase service utilization and access for impoverished women, individuals with lower levels of education, and residents of isolated rural areas. Furthermore, context specific information pertaining to cultural barriers and political ecology are required.

**Supplementary Information:**

The online version contains supplementary material available at 10.1186/s12913-024-11074-0.

## Background

Over the last few decades, the issue of equitable distribution and utilization of healthcare services has captured the attention of scholars, governments, and policy makers. While some countries have made much improvement in this regard, others have lagged behind. Child healthcare service use is no exception, and it has recently become a global concern [1].

Equity in health and health service utilization is a focus in the global health discourse as one of the cornerstones of primary health care (PHC) [2]. The United Nations has set a goal to reduce global neonatal deaths from 25 per 1,000 live births to 12 per 1,000 by the year 2030 [3]. Achieving this target will require strong commitment from both service providers as well as financiers of the health sector, including government and community leaders [3]. Among the World Health Organization’s main focus areas for under-five health, is the reduction of inequities in accordance with the universal health coverage principles. This includes addressing the health needs of children in poor and remote communities. Many countries have implemented various programs aimed at minimizing unnecessary disparities in health service utilization [4]. Community based health services in particular have been found to be effective in minimizing inequities in health status and health service utilization [4]. For instance, the Ethiopian government is committed to improve equity through the health extension program and other initiatives [5]. Moreover, Ethiopia included the equity objective in its health sector transformation plan [6]. Nevertheless, inequities in service coverage and difference in maternal and child health outcomes remain a challenge [7, 8]. The coverage of child health services and basic child immunizations has favored wealthier, more educated, and urban populations. [9]. For instance, despite significant decline is observed in under five mortality from 123 per 1,000 live births in 2005 to 59 in 2019, still there exists a disparity between different population groups [9].

Ethiopia met the MDGS for child mortality rate (CMR) in 2013 [10]. However, the gains made between 1990 and 2013 were not uniformly distributed among Ethiopians; inequity indicators of mortality by wealth had not significantly decreased. During this 23-year period, the mortality among the poorest was unchanged. Even though child health services are supposed to be provided free of charge at public facilities, the disparity in access to or utilization of the services is high in Ethiopia. Like mortality disparity, there is a considerable disparity in coverage of life-saving interventions by wealth status and place of residence [11].

Addressing equity is a significant challenge in healthcare delivery in Ethiopia. The barriers that were reported to be significantly associated with service utilization included geographical access as a function of distance; financial barriers; and socio-cultural factors such as language, cultural norms, health beliefs and perceptions, maternal education and decision making power and lack of knowledge and awareness, which in the aggregate can lead to low demand for and use of services, particularly by the poor [12, 13]. Long distances and extended travel times remain key barriers to access health facilities in many rural communities in Ethiopia [11, 14]. For instance, in Indonesia proximity to healthcare facilities significantly decreases child mortality [15]. Furthermore, according to a study from Uganda, Nigeria and Ethiopia long distances to health care facilities cause delays seeking care [16–18]. Even where health care services are available, the cost of seeking care may delay or prevent poor households from accessing them. This problem is particularly discriminating in rural areas where the density of modern health care facilities is low and in settings where transportation systems and road infrastructures are poor [18]. Furthermore, over the last five years, Ethiopia has faced internal conflict and political instability which exacerbated inequity in the utilization of child health services among the poor and in rural communities. Both insecurity and scarce resources are critical issues in child healthcare accessibility for women living in conflict zones and socioeconomically unstable settings [19].

Political instability disrupts electricity, water, and food supplies, destabilizes social and welfare systems, including the health and transportation systems, and increases unemployment, homelessness, and poverty—all of which have a negative impact on the use of maternal and child health services [20]. Hence, these issues did not addressed in any of the studies done so far.

Thus, while many studies have been conducted on the utilization of healthcare services, there is insufficient comprehensive evidence on the barriers of equity in accessing and in utilization of healthcare services for under-five children from policy makers and community level perspective. Therefore, the objective of this study is to examine the context of specific barriers to achieving equity in child health-care services utilization in Ethiopia.

**Fig. 1:**
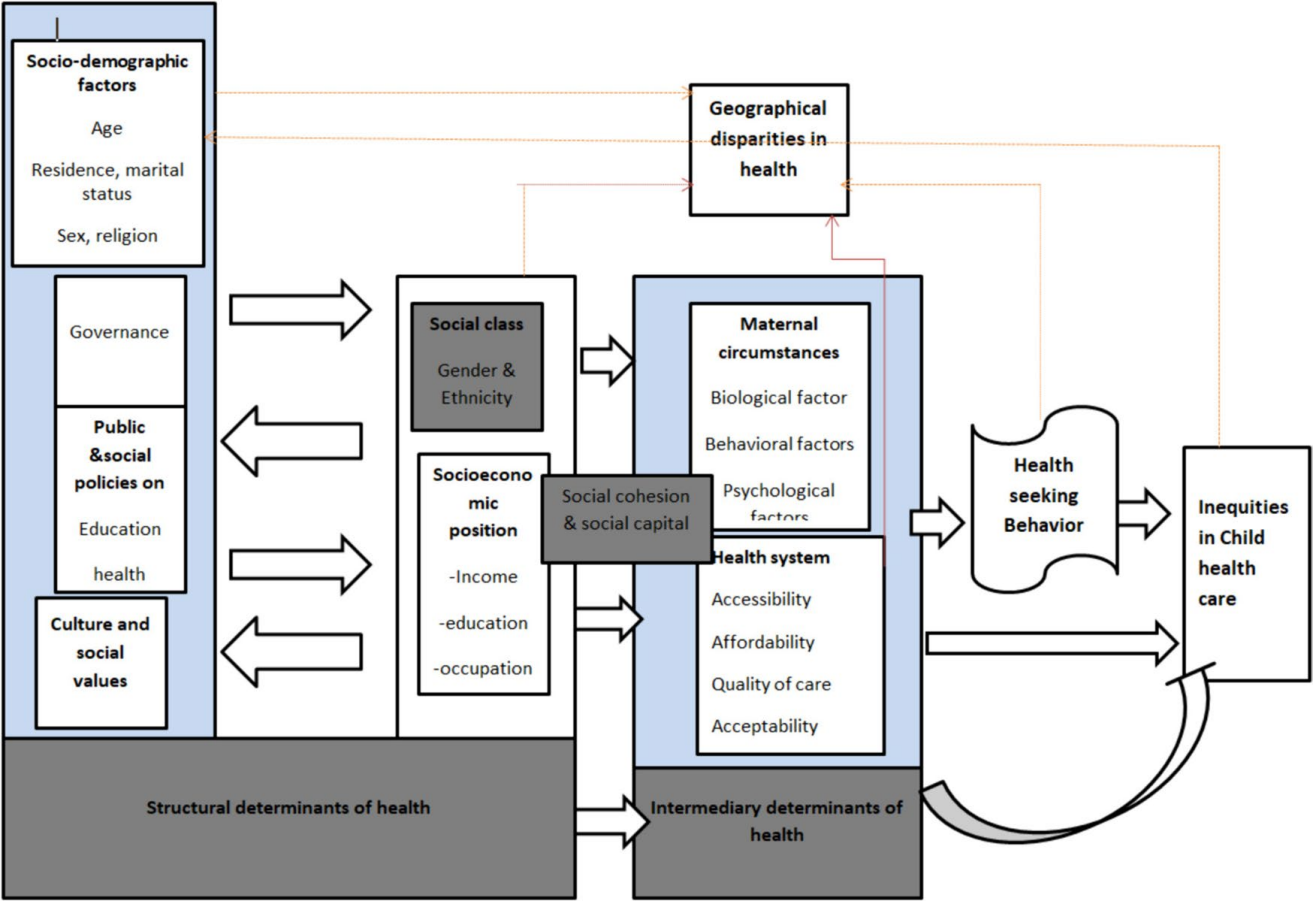
Modified Andersen and WHO conceptual framework, on social determinants of health inequity

Inequity in child health care service is a major public health problem in developing countries, including Ethiopia. Accordingly, the study explored barriers to equitable healthcare services for under-five children, their health seeking behavior, geographical variation, their awareness, perceptions, attitude and political impact and policy contents of the country. The findings will benefit program leaders, policy makers on health inequality reduction and serve as an input to policy documents related to the new health sector strategic plan. Moreover, mothers and under-five children’s are directly benefited from the finding. Conceptual framework shows how different barriers affect equity in utilization of child health services (Fig. 1).

### The study setting and approach

**Fig. 2:**
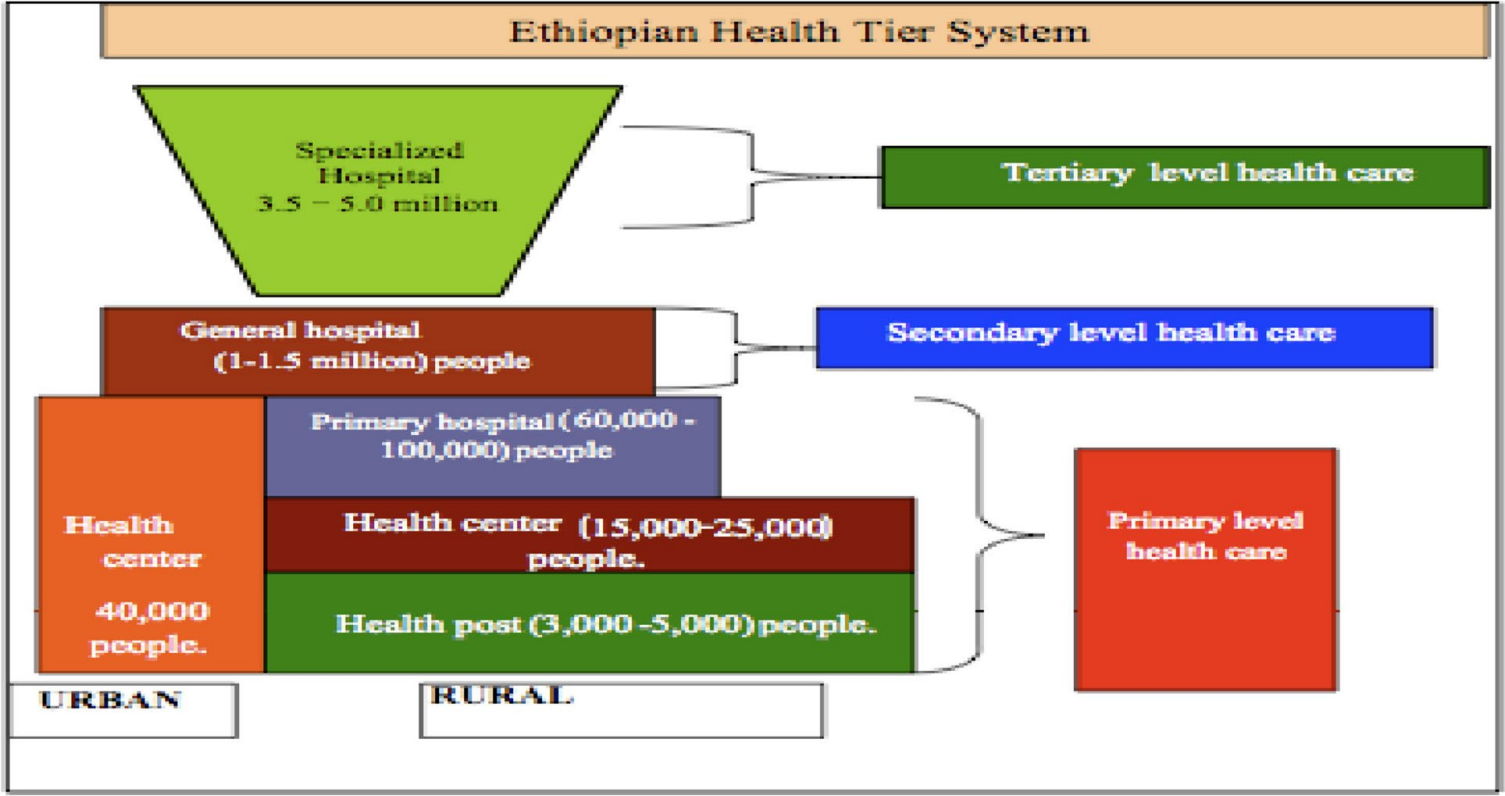
Ethiopian health care system [17]

This study was conducted in Oromia Region, Arsi Zone, Zuway Dugda District from June 1–30, 2023. The Ethiopian healthcare system is three-tiered, comprising primary, secondary, and tertiary care [Fig. 2]. The primary level healthcare system is responsible for providing child health services, such as immunizations, and the treatment of sick children. The primary care unit includes primary hospitals, health centers, and health posts which are responsible for providing services to rural communities ([17]; Arsi Zone Health Department report, Unpublished data, 2022). Women's development armies (WDAs) provide support to health extension workers (HEWs) by organizing and connecting women and their children with healthcare facilities. Based on 2022, the Arsi Zone report, Zuway Dugda district was low in utilization of child healthcare services and the population is low in socioeconomic status and mostly depends on the Safety Net program for nutritional and financial needs (Arsi Zone Health Department report, Unpublished data, 2022). The goal of the Safety Net program is to preserve family assets while generating new ones for the community. To achieve this, the program offers food or cash incentives in exchange for public works projects that improve the environment or create local infrastructure, like roads (e.g. terracing).

### Participant selection

The study includes 20 in-depth interviews of key informants (KII) and six focus group discussions (FGD). By taking into account various factors that contribute to variations in the use of child healthcare services, study participants were selected from a variety of demographic subgroups. The study participants were drawn from different segments of the population by considering different dimensions that explain disparities in utilization of child healthcare services. The selection of participants was based on their experience of child healthcare services as well as the information they possessed. For the purposes of this research, to ensure representativeness, and to understand the multifaceted levels of the study framework within society and how individuals and the environment interact within a social system, we used maximum variations sampling technique and we classified the participants into four groups. They were “mothers or caregivers who have under- five children”, “males who have under-five children”, “healthcare leaders at different levels” and “healthcare providers at different health facility”. The first group,, “women” refers to mothers who were gave birth prior to the study period and currently having under-five children. The second group were “males or husbands of the women who have under-five children”. The third groups, “healthcare leaders” like; heads of the health centers, district health office, expertise working on child health programs in district, Zonal, regional or national level. The fourth group, “health-care providers”, refers to health professionals, including doctors, health officers, nurses, midwives, and health extension workers working at different health facilities in Arsi Zone and having direct relation with child healthcare services.

Participants in the focus group discussions (FGDs) could be women and their partners who had under-five children at the time of the study. The participant mothers or caregivers were recruited by the HEWs and *kebe*le (neighborhood associations) leaders. They were identified on a purposive basis with the help of health extension workers and were contacted a few days before the planned FGD to explain the objectives of the study and request their participation. For the key informant interviews, the study participants were contacted by the principal investigator two weeks before the interviews. The information was collected based on the principle of saturation; for our case at least 16 interviewees were needed to reach information saturation principles. Then, data collection was terminated when no new information was generated.

### Interviews

An interview guide was prepared for both the key informant interviews (KII) and FGDs. First, the guides were prepared in English language and then translated into the local language [21]. Then, the guides were pre-tested and problems relating to the sequence of questions, conceptually similar questions, and sensitive wording were corrected. The data collectors for the KII and FGD were professionals with the background in health and health related fields with master and who are experienced in collecting qualitative data. Moreover, they are fluent in the local language and familiar with the culture of the local community. Key informant interviews were conducted at the office or at the health facility where the interviewee worked and FGDs were conducted in community halls or public rooms. Both key informant-interviews and focus group discussions were audio-recorded. Additionally, complementary observations and notes regarding the remarks made by the participants and their interactions were made.

### Data analysis

The principal investigator and the moderator transcribed each interview and FGD word-for-word in the local Afaan Oromo and Amaharic languages and then translated the transcripts back into English. The translations were verified by listening to the recordings while re-reading the transcripts. The data were analyzed thematically using NVivo 12 software and narrated in the pattern linked to child health service utilization. Major themes representing the FGD participants and in-depth interviews are presented in the findings section, with illustrative quotes included to support the main findings.

### Trustworthiness

In qualitative research, trustworthiness is determined by credibility, dependability, conformability and transferability. Establishing credibility involved the primary researcher spending a considerable amount of time at the study site to get a feel for the environment, receiving ongoing feedback from peers during peer debriefing, and applying negative case analysis. Dependability was demonstrated by providing an in-depth explanation of the techniques employed, keeping careful interview records, and recording the analytical procedure. All events that took place in the field, the researchers' personal reflections on the study, any phenomena that emerged during the investigation, and pertinent details of their personal histories were documented in order to verify that the interpretations of the findings were derived from the data and were not the product of their imagination. The investigators attempted to build rapport and trust with the informants by developing a long-term attachment because they were skeptical or doubtful if the information felt off. Triangulation of data sources was also employed. A thorough description that includes explaining each step of the research process was employed to aid in the transferability of research findings. At the end of each qualitative data collection session, the data collectors rephrased the collected information by summarizing major points and obtained approval from the participants for the corrected summary.

## Findings

### Characteristics of the study participants

A total of six focus group discussions (FGD)- three with mothers and three with fathers of under five children were conducted. And 20 key informant interview (KII) were held. The number of FGD participants ranged from 8 -12 in each groups. The majorities of women’s participating in FGDs were housewives and had at least one child under the age of 5 years in their care at the time of the FGD. The key informant interviews were conducted with leaders and policy makers at different levels of the health care system and a healthcare worker, including FMoH child health directors, Regional Health bureaus experts, Zonal Health office child health experts, woreda health office heads, Health center heads and health extension workers at health posts were involved. In all, 28 men and 30 women took part in the FGDs. In contrast, six HEW, three heads of health centers, one head of the district health office and with ten experts participated in the key informant interview. Each FGD took on average 42 min (38–52 min), while the key informant interviews took about 36 min (19 to 55 min) (Tables 1 and 2).

**Table 1:** Basic characteristics of the participants in the in-depth interviews

KII Code	Age	Educational status	Marital status	Religion	Responsibility related to Child Health
KII-01	45	MPH	Marrid	Protestant	Expertise
KII-02	30	MPH	Single	Orthodox	Expertise
KII-03	46	MPH	Married	Protestant	Expertise
KII-04	44	MPH	Married	Protestant	Expertise
KII-05	39	MPH	Married	Orthodox	Expertise
KII-06	40	MPH	Married	Protestant	Expertize
KII-07	45	MPH	Married	Proestant	Expertise
KII-08	55	MPH	Married	Protestant	Expertise
KII-09	39	Level 4	Marred	Muslim	HEW
KII-10	35	Level4	Marred	Muslim	HEW
KII-11	48	Level 4	Married	Muslim	HEW
KII-12	30	Diploma	Married	Muslim	Head of Health Center
KII-13	28	BSc	Married	Orthodox	Expertise
KII-14	41	Diploma	Married	Muslim	Head of Health Center
KII-15	28	Level 4	Married	Muslim	Head of health center
KII-16	28	Level 3	Divorced	Muslim	HEW
KII-17	34	Level 4	Married	Muslim	HEW
KII-18	32	BSc	Married	Orthodox	District health office head
KII-19	40	BSc	Married	Orthodox	Expertise
KII-20	29	BSc	Married	Protestant	HEW

**Table 2:** Basic characteristics of the focus group discussion participants

FGD Participant			
**FGD No**	**Category**	**No of Participants**	**Site**
FGD-1	Female FGD	10	Ogolcho rural
FGD-2	Female FGD	10	Haraxa rural
FGD-3	Female FGD	10	Gulele rural
FGD-4	Male FGD	12	Ogolcho town
FGD-5	Male FGD	8	Haraxa town
FGD-6	Male FGD	8	Ula arba

## Barriers to equitable healthcare services for under-five children

### Six major themes emerged from the findings

These include; barriers related to low awareness, low *socioeconomic status, geographical inaccessibility, barriers related to deficient healthcare system, cultural and behavioral constraints, and political instability and conflict, all of* which lead to unmet healthcare needs such as delay in receiving appropriate care and inability to obtain healthcare services (Table 3).

**Table 3:** Barriers of equity in health care service utilization for under-five children under six them emerged from in-depth interview and FGD

Themes	Sub-themes/ Category	Codes
Lack of awareness of the service	-Lack of information about availability of services -Low awareness -Lack of knowledge about benefits of child healthcare	- Lack of adequate information (5) -Lack of or limited knowledge of benefit of child healthcare (4) - Limited health literacy (7)
Socioeconomic barriers	Low household income -Low educational attainment	-Financial barriers of families (6) - Cost of care and transportation cost (9) -Indirect costs health care fee (4) -Women’s education (3) -Paternal education (5)
Geographical barriers	-Distance of health facility from home - Lack of roads -Poor roads - Transportation problems -	-Lack of proximity of the health services from home (16) -Unavailability of transportation system (18) -Barriers in reaching health facility (6) - Transportation related barriers (12) -Lack of ambulance services (150 -Weather conditions and poor roads (3)
Health system barriers	-Unavailability of the services -Inaccessibility of the heath facility -Unaffordability of health services cost - Absence of compassionate and respectful care among health professionals - Unavailability of drugs and medical supplies	-Inadequate material, drugs, laboratory supplies (8) -Distance from home (12) - Payment for ambulance (4) -disrespectful and abusive care by HCWs (4) -Lack of experienced health providers (2) - Language barrier (2) -Low quality of care (2) -Lack of transportation (12) -Lack of planning for marginalized peoples (7) - Lack of giving priority for poor people (5)
Cultural and behavioral barriers	- Traditions and beliefs -Taboos related to neonatal death -	- Cultural beliefs and traditions prohibit the neonate from accessing health facility (8) - Traditions and beliefs (7) -Lack of women’s autonomy (5)
Politics, conflict and security issue	-Insecurity due to conflict - Political instability	People are killed (22) Closed roads (19) -No transport (20) Health facilities are not functional or destroyed (14) -Health professionals feel insecure or are killed (18)

#### Lack of awareness about benefits of the services

Lack of awareness and misconceptions were one of the top reasons raised by KII and FGD participants for not using healthcare services for under-five children especially in rural communities. Recognition of illness and the potential benefits of treatment are pre-requisites for health care demand. Communities who lived in remote areas and are undereducated tend to have little knowledge concerning health issues. Rural people have insufficient exposure to the media, attending low level of schooling to grasp and understand health related information. The key-informant interviewees and FGD discussants reported that because of low health literacy, rural community and the poor households have less access to health facilities to get treatment for childhood diseases, and for immunization services. One of the key informants mentioned that there are variation or differences among urban and rural rich and poor, literate and uneducated people in child health care service utilization.

“…*. Children who visited our health center with malnutrition were from remote and far to reach areas and were brought to our health facility only after these cases were seriously complicated. So there is great variation among urban and rural, rich and poor, literate and illiterate communities in child health care service utilization in our district*.” Male , KII, age 34years

One FGD discussant from women group added her experience and her awareness of immunization and availability of free service in health post in such ways;

*“Yes, if I had been aware of the benefit of immunization and informed that they were given free of charge, I would have used these services for my sick child from health posts, not from traditional healers”* Female FGD discussant, age 35 years, Seeking care from traditional healer

The health extension workers at health posts also approved the lack of awareness among mothers and caregivers on the availability of health service which jeopardizes health- seeking and utilization of health service for their under-five children. One worker said that.

*“Most of the women’s and care givers did not know about the availability of treatment at the health post, especially for diarrhea and pneumonia. Those women’s who live near a health facility, are educated and young have more awareness about childhood illnesses and seek care from health posts than uneducated mothers; this may result in inequitable utilization of health services by illiterate care givers*” KII, Female 35 years

In some areas there is a mix of knowledge about utilization of healthcare services for under-five children. Health professionals used *abbaa gadaa, or hadha sinqee* (male and female cultural leaders) and members of the female development army (FDA) to raise the level of awareness in the community. One key informant interviewee shared the experience of his districts in utilizing women’s development army and these cultural leaders to increase the knowledge of the community as follows;

“We improve the awareness of our community on child healthcare utilization through women’s development army and cultural leaders, we trained these women about early recognition of maternal and child health danger sign. we provide them local COC for them. By now in our district, women’s development armies have equivalent knowledge with HEW and, we used them to teach the community”. Male, Key informant, age 42 year.

#### Socioeconomic barriers

Lack of sufficient income at household level and low level of maternal and paternal education were identified as major barriers for equity in utilization of healthcare services for under-five children. As part of its HSDP II strategic objectives, the Ethiopian Government intends to address equity in maternal and child health, particularly for the impoverished and rural communities, by providing free health services to these subgroups and allocating a sufficient budget. However, the actual and perceived cost of seeking care keeps some people from traveling to medical facilities. Out-of- pocket costs of health care, cost of transportation and living cost may prevent poor people from using services, leading to untreated childhood illness.

For instance after they reached to health facility, they obligated to pay for medical treatment or drugs they used to treat their children. In this case some advanced diagnosis and treatment is not available in governmental health facilities.. For example, CT scan and MRI to diagnose severe childhood diseases and some essential drugs to treat pneumonia, sepsis and diarrhea were not available in health centers and in health posts. They were advised to get this treatment from private clinics and to buy the drugs from private pharmacies. However, or mothers could not afford to purchase them from private clinic.

A woman from FGD discussant explained her experience of an availability of certain services in Government health facility and high cost of services in private clinic as follows:

“Yes, nowadays, the cost of drugs and treatment for childhood illness is increasing, when I used to get treatment for my sick child from a health center or health post the health professional referred me to a private clinic to be seen or diagnosed by a highly expensive machine; I am unable to afford for this machine. Moreover there were no drugs at the health post and the health center. They told us to purchase them from private clinics. So, how can the poor people get treatment from Governmental health facility?” Female FGD discussant age, 34 years, with low income.

Another FGD discussant described this problem as follows:

“Yes, getting treatment in this health facility is good but sometimes you go here and there to get examined and prescribed for drugs and you need money for those drugs. If you don’t have money, then you remain with the illness” Female FGD discussant, age 29 years.

The study participants suggested that, socioeconomic healthcare inequity must be addressed by healthcare system revisions such as the provision of health insurance, fee retention; waiving and exemptions from fees for poor people, and subsiding the cost of the transportation were considered as solution to reduce inequity in health care services.

#### Geographical barriers

Distance of health facilities from home and unavailability of motorized transportation were another major barrier to health services utilization. Pit the fact that availability of some community based services should increase health service utilization to caregivers, distance from homes to health facilities, poor roads and unavailability of motorized transport were major barriers for many people. Distance from health centers and health posts and lack of transportation and cost of transportation were cited as barberries of equity for child health service utilization by rural and the poor communities. Long distances, shoddy road construction, and a shortage of ambulances make it difficult for residents of remote communities and low-income families to get to medical facilities and thus have fewer opportunities to vaccinate their children. One key informant said that.

“…the primary issue facing this district is the lack of transportation and the distance between the residential area and the medical facilities*. The caregivers were unable to get transportation service easily. In some areas the distance between health facilities and residential areas of the community is too far, besides there is no road to get access to health facility. We need more vehicles at health center level; moreover, the transportation issue cannot be solved unless quality roads will be constructed for the community.”Male, key-informant interview, age 40 years.*

Another FGD discussant said that.

“ Yaa, we move more than 30 km on foot to access health facilities, especially health centers, there is no road for cars., we carry our sick child on our backs to get treatment from this health facility” FGD, Male, age 44 years.

One FGD described the transportation problem a follows:

“…even though roads were constructed, there is no reliable transportation system in our area. Ambulance service is not available in our area, no mobile network to call to ambulance service. Moreover, if we were hardly access the ambulance, we are requested to pay 1000 Birr for fuel. Therefore, the Government and concerned body has to understand and solve our situation related to distance and transportation problem.” Male, FGD discussant, Age 49 year.

The study participant also suggested that geographical and financial accessibility barriers have to be addressed by bringing services closer to homes or residential areas.

#### Healthcare system barriers

Certain aspects of healthcare system were identified as barriers to equitable healthcare services for under-five children. In Ethiopia, important deterrents include unavailability, unaffordability of the service, and closure of health posts during working hours and issues related to behaviors of the health professionals were the emerged theme from this study.

One of the important barriers of equity in utilization of child healthcare services especially by poor were unavailability of child care services at health posts. Even though the health posts are supposed to give services for the rural and poor populations, it was closed on many working days and at weekends. In addition, absence of health extension workers from the duty during working hours, services inconsistently and unavailability of drugs in the health posts were barberries of equity raised by KII and FGD discussants. One of key informant interviewees explained his observations as follows;

“Even though, the health posts are expected to give maternal and child health services for the rural community free of charge, how the poor and the rural community get these services, the health posts were closed during working hours, most of the time the HEW workers are in another duty, they were assigned to collect taxes and insurance from the community, so the richest household will get these services from private health institution but the poor and the rural community is in problem in accessing these services” Key-informant interview, Male, 45 years.

Besides giving health services, in some rural areas the health extension workers are assigned to other administrative and political activities. A health extension worker in health post acknowledged the absence of health services during working hour in such ways;

“ How can we give health services for the poor community, we are assigned to collect insurance, taxes and to register member for the political parties, if we say no we will be fired, most of the time the health posts were closed, all services were intercepted, mothers from rural area repeatedly came for immunization, but they did not get us in the health post, those mothers who were educated and have the money for transportation may went to health centers and Hospitals to get immunization service, but the poor mother were waiting us till the health post is opened” Female, Age 39 year.

One woman from FGD participant also explains her experience as follows;

“One day my 3 years old child was sick and I came to consult the HEW, but, the door is closed and she was not around” Female, FGD discussant, Age 38year, rural community

Another important finding from this qualitative study was issue of marginalized populations. The health services do not cover marginalized and poor people, like, beggars, around churches, mosques and along roads on child health services especially immunization**.** Key informant participants from the one *woreda* health office described this issue as follows;

“Here is the gap, now the health facilities have no plan and willing to give immunization services to marginalized poor people like; beggars around the mosque, church, and on roads. These poor people are totally forgotten, the motivation of health workers to serve this community is almost zero or near to nil. All vaccination mandates are given to HEW, but now health centers and health posts are not connected to these people and their children’s are not vaccinated at all. There is no supervision or support from higher officials, no accountability among HEW “KII M ale, 45yer.

Lack of adequate supply of medicines and other medical supplies emerged as a recurring theme in FGDs and KII at both the policy and service delivery levels. The health posts do not have all basic medicines available and end up giving inadequate drugs, no separate budget is allocated for child health by Ministry of Health or the regional health bureaus. Donors, NGO,s and partners have reduced their budgets and support of child health programs.

One KII participant shared his perceived cause of inadequate supplies and budgeting for health facility as follows:

“…Currently only limited budgets are allocated to the health sector, especially for maternal and child health. There are no donors and partners who support the healthcare system; this is probably linked to the current Ethiopian political upheavals. This creates problems for free services for maternal and child care. In my opinion this is the cause of an availability of materials and some drugs at health facility” key-informant-interview, age 44 year.

Disrespectful care and treatment was the issue raised as barriers to equity by caregivers for their under-five children. Ethiopian communities pay attention to respectful and quality of care, therefore giving preference to urban health centers, which generally meet patient expectation. But urban health facilities also discriminate against poor people. A female FGD discussant raised the issue of non-compassionate and disrespectful care given to her at an urban health facility, as follows:

“Yes, we looked unclean and came from rural areas, the health professionals treated us as not as humans and gave us poor care. They did not touch us by their hands or used apparatus to examine our problem. They simply asked us about our illness and gave us prescription to buy drugs” Female, FGD, 42 years.

Respondents suggest that, the government need to ensure the availability of adequate essential vaccines, drugs and supplies in health facilities. The FGD discussants further emphasized that, both central and local healthcare systems need to allocate adequate financial resources and procure adequate logistic and material supplies towards effective implementation of quality healthcare services.

#### Cultural and behavioral barriers

Low demand and utilization of modern health interventions often derives from deep-rooted attitudes that reflect culture, social norms and traditions of the community. Few FGD participants mentioned that cultural barriers such as using traditional medicines at home and taking the children to traditional healers were barriers to using child health services, especially in rural areas. In some areas peoples believed that the cause of the illness is caused by supernatural agents, exposure to cold, wind or the devil eye. Therefore they do not bring their children to health facilities. Many poor mothers and care givers in rural areas use traditional medicine or religious interventions such as payer as the first treatment for childhood illness because of their ready accessibility and low cost, as stated by one father:

“I have encountered people in some districts who delayed treatment because of traditional beliefs. One of them said … If my child gets sick, I will not bring it to a health facility immediately, I will wait until the disease matures and shows full blown sign can l be observed or till it will resolved by itself” key-informant interview, Male 42 years.

There are also other traditions, customs and beliefs among some rural communities which are barriers to equity of child health services. For instance *haamachisaa* is a kind of blessing used as the first treatment by traditional healers for neonates aged less than 3 months before seeking care services from health facilities. They believe that *haamachisaa* prevent malicious birds or the evil eye to inflict illness on neonates, as described by one mother:

“ in our area some of the rural communities will not send their** “**children below three months of age” to get immunization services from health facilities before they practice haamchisaa or blessing services from a traditional healer because a bird or the evil eye may see the neonate “ Female, key- informant, 39 years.

In some rural districts, obstacles to child health care service utilization include the use of traditional uvulectomy, getting treatment for measles from traditional healers and using holy water *(tsebel)* at churches when children fall ill.

“In our area, when their child develops measles some of them refuse to take their children to health facility because they believe that the treatment there will cause girsha, the dissemination of the rash to different organ systems” Male, key-informant, 30 years head of HC.

Another FGD discussant described her preference of traditional healers for her sick child because of cost of the drug as follows;

“I visited a traditional healer for my child when he had tonsil, because drugs and repeated treatment from a health facility are expensive; After the tonsils are removed by a traditional healer there is no recurrence, so it is less costly for me” Female, FG, Age 40 year.

In another way less attention was given for morbidity and mortality of the child by rural community, especially to the neonate (if a neonate died) the funeral ceremony will not be practice in the church or mosque. The burial or funeral ceremony is accomplished at near house of the parents; the dead body is not brought to church or mosque. The community did not consider a neonatal death as a death of human being or adult death but, is concealed, as described by a male key-informant:

“ Here in the community less attention is given to child health, especially for the newborns; if the newborn dies the dead body will not brought to a church or mosque but it will be buried around the home. Nobody will go to that home to morn with the parents” Male key-informant, 42 years.

In many Ethiopian communities, women’s have low autonomy to decide for her own and their children’s health in Ethiopia. They need the permission of their husbands to seek care for their children, because of economic, psychological and material dependence. The norms and values of the community also reinforce this behavior.

One of the important finding of this study was inequity related to ethnicity. Almost all KII and FGD participants stated that there is no disparity in healthcare service utilization because of ethnicity.

“…..Even though Ethiopia is having a diversified ethnic group still there is no marginalization or inequity in utilization of child health services from health facility because of ethnicity; rather they encounter barriers related to language in understanding and to get consultation from service providers” Key-informant, Male, Age 39 year.

The study participants further suggested that barriers related to health illiteracy or mistrust of the healthcare system have to be addressed by involving different stakeholders such as community leaders, traditional healers and religious leaders**.**

### Politics, conflict and security issues

Over the last few years, Ethiopia is suffering from different types of military conflicts between the Ethiopian government and insurgent forces in most regions and administrative areas. This protracted conflict hinders maternal and child health service delivery affected communities, especially in isolated rural areas. As a result, health services could not operate safely in the war zone, Increasing the incidence of vaccine-preventable diseases and malnutrition. A male FGD discussant explained the effects of conflict on maternal and children service utilization as follows:

“ In our district there is continuous military conflict between the government and rebel forces; most of the time the health facilities were closed, there is diversion of supplies for maternal and child health services to the armed forces, no immunization services was given to the community during this conflict period, roads were closed, the health professionals fled health facilities because they felt insecure, even ambulances assigned to MCH services were used for military purposes;, the rich may get the service from private clinic, the poor did not get anything, simply waiting an interventions from God,, or simply wait to die or migrate to other places” Male, FGD, age 45 years.

One key-informant interview participant reported his observation of security problem on child health services in his district as follows:

“Regarding the issue of security problem, currently in our area there is a military conflict between government and rebellions. Due to this there is no maternal and child healthcare services, 24 h ambulance was served for political purposes, as a result mothers and children are dying from severe anemia and severe pneumonia at their home, therefore, politically instability and conflict among Government and armed rebellion force exacerbate the existed disparity in utilization of healthcare services for mothers and children in our district”. Key –informant, male, age 41 years.

The research participant added that communication between opposing groups is necessary to resolve political unrest and conflict which has direct impact on child healthcare utilization.

## Discussion

This study aimed at exploring barriers of equity that mothers and their children face in accessing and utilization of healthcare services for under-five children. The findings point out multiple dynamics of barriers of equity to care-seeking and utilization of healthcare services in Ethiopia.

In this study the barriers and challenges linked with access and utilization of equitable healthcare services for under-five children were found to fall under six themes***;**** lack of awareness about availability of the service, socioeconomic barriers, geographic barriers, health system related barriers, cultural and behavioral barriers and political instability and military conflict related barriers.* These barriers are inter-related and complex in nature. As key-informants and FGD discussants reported that lack of awareness was one of the top barriers for not using healthcare services for under-five children, especially in rural communities. Populations who have settled in far to reach areas and uneducated have no equal awareness about health related issues compared to urban and well educated populations. Their reasons are people leave in rural area has insufficient exposure to media, attending low level of schooling to grasp and understand health related information. Most studies reported that because of low health related literacy, rural community and the poor household had delayed to access health facility to get treatment for most of childhood illnesses, and vaccination services [22]. In this study having participants confirm that there is a gap in knowledge regarding the causes of childhood illness and regarding the availability of treatments at health posts, it is clear that a campaign to educate and mobilize community members will be necessary. The need for transmission of information about the availability of services was also highlighted by several other studies [23]. Studies in Ethiopia reported that, HEW home visits were reportedly valuable for increasing awareness and use of services and mothers of under-fives who received health information [24]. Different studies suggest that community education and mobilization campaigns may increase level of awareness of communities. One study also reported that HEWs and HDA were credible sources of health-related information [25, 26]. For example, the HEW increased the awareness of communities during pregnant woman conferences, vaccination campaigns, and other community meetings.

This study further highlights that, socioeconomic barriers to health care utilization are strong deterrents that increase under-five mortality in Ethiopia. Limited financial resources for medical treatment and low educational level of parents are barriers to the use of healthcare services for children among disadvantaged populations. In this regard, the Ethiopian government plans to provide free health services for women and under-five children, through the HSTP. II but our finding revealed that low household income, low level of maternal education, and out-of- pocket payments for health care prevent poorer people from using services for under-five children. Furthermore it should be noted that the government of Ethiopia has adopted a waiver fee policy for the vulnerable groups. However, cost of services still play a major role in access to service since the exemption from paying for the services is unevenly applied.

Several studies corroborate our findings of the socioeconomic impact on health service utilization [27, 28]. A study conducted by Daniel et al. confirmed that the levels of household income and health literacy affect access to healthcare services [27]. Moreover, indirect costs such as loss of work time, loss of income and transportation cost have a significant economic impact on poorer families [28]. Implementing health insurance scheme and waiving user fees may shield the poor from these charges and weaken household health budget constraints.

Our findings of the negative impact of low education of parents is corroborated by several studies. [29, 30]. Pregnant mothers with higher education are more aware of the significance of good nutrition and child care as well as the prerequisites for being healthy [31]. Education plays a vital role in shaping attitudes, opinions, customs, and norms and also promotes the adoption of new ideas and values.

The result of our study revealed that, distance from health facility, lack of transportation and uncomfortable road topography especially in summer were mentioned as an important barriers of equity in healthcare service utilization for under –five children. Previous studies in Ethiopia confirmed that far to reach regions, districts and areas often face special issues and problems compared to non-far to reach areas [32, 33]. Several studies in other countries also showed that travelling to a health center was challenging for caregivers of children residing in far to reach areas and cost of transportation, unreliability and its unavailability of services were the main impairments of equity in accessing healthcare services [34–38]. For example, a study of measles vaccination coverage in various African countries found that distance was a key factor in determining the level of immunization coverage [39–41]. In addition to the inverse relationship between distance and health services utilization, geographical location of health facilities in isolated rural areas also jeopardizes the staffing of health facilities. Doctors, midwifes and nurses are less eager to serve in such areas than in urban communities and vaccines and flooding may prevent the delivery of vaccines and drugs to distant mountain communities during the rainy season.

Respondents highlighted the need to ensure reliable availability of HEWs at the health post during opening hours and extending the hours of the health post so that services would be available working hours and on weekends. Such closures have been shown to be a major challenge in previous studies [42]. HEWs travel for activities such as collecting taxes registering political membership from the residential and payment for health insurance from the community should be stunned by the community and so that there is at least one HEW in each health post to give services for the community**.** Another important finding from this study was issue of marginalized populations. Some key-informant and FGD discussant cited that, the health facility is not have especial plan to address the services to marginalized poor people, like, baggers around the church, mosques and around roads on child health services especially for immunization services. Hence this may create critical inequity in child healthcare service utilization among the poor.

Barriers to equity in access and utilization of services extend beyond accessibility and availability issues, disrespectful care and negative attitude acts as a barrier to accessing health care services. Negative attitude of health workers in the form of verbal expression, represented a theme of recurrence as a barrier of equity in utilization of the services. Female FGD discussant raised the issue of non-compassionate and disrespectful care given to them by health professionals at health facility and they were receiving poor quality of care, and there is no companionate care for the poor. Improving quality and outcomes at health centers offers an incentive for the utilization of a service. In many African countries, low quality of health services has been identified as a hindrance to equitable access of services [43]. In the current study, few participants mentioned that cultural factors like home remedies taking the children to traditional healers were obstacles to utilization equity. Other studies from Sub-Saharan Africa show similar results [44]. This shows that, traditional beliefs and norms of the community impede from seeking-care modern healthcare and utilization of the services from health facility.

One of the promising finding in this study was, even though Ethiopia have a diversified ethnic group there is no report related disparity or inequity in utilization of child health services because of his/her ethnicity, rather they encounter barrier related to language in understanding and to get consultation from service provider.

Our finding revealed that, war and political instability disrupt health services accessibility and utilization. There was also reported from several other countries, including Afghanistan, the Democratic Republic of Congo, Pakistan, and Somalia [45]. Key impacts include disrupted infrastructure and supply chain; violence against health workers; difficulties retaining health workers; delivery service interruptions; and displacement and migration [46]. For populations affected by military conflict, adopting flexibility surrounding age and eligibility criteria can increase immunization coverage.

### Strength and limitation of this study

Strengths of this study include the collection of data by experienced interviewers, efforts made to increase trustworthiness of the study, checking transcripts against audio-records and field notes by two independent experts, and use of the participants’ own language for data collection. In addition, inclusion of participants from all levels of the healthcare system and caregivers (both mothers and father) of the children broadened the range of experiences and opinions on inequity in child health services accessibility and utilization. The major limitation of this qualitative study is that its findings are may not be generalized to other settings. Furthermore, since the study was only conducted in one district, it might not be representative of the entire nation.

## Conclusions and recommendations

We conclude that inequity in child healthcare utilization continues to be an important challenge confronting Ethiopia. Constraints such as poor community awareness of the availability of curative healthcare services, geographic inaccessibility, inadequate healthcare resources, socioeconomic barriers, and constraints related to the functioning of the healthcare system and political instability and military conflict were the most cited barriers to equity.

To achieve equity, Ethiopian policymakers and partners need to invest in health infrastructure, including bringing services closer to people by constructing new health posts, health centers and roads in rural areas, and increasing the quality of services. In addition, context-specific cultural barriers such as the use of traditional medicines and illness beliefs need to be addressed through health promotion and military conflict needs to be solved through dialog between opposing bodies.

### Supplementary Information

Supplementary Material 1.

## Data Availability

The data that support the finding of this study are available and attached as related files.

## Abbreviations

COC: Certificate of competency
CRC: Compassionate respectful care
FGD: Focus group discussion
FMoH: Federal Ministry of Health
HCWs: Health care workers
HEW: Health extension workers
KII: Key-informant interview
MCH: Maternal and child health
MDGs: Millennium developmental goals
ORHB: Oromia Regional Health Bureau
PHC: Primary health care
UN: United Nations
