# Supplementary material for: Barriers to equitable healthcare services for under-five children in Ethiopia: a qualitative exploratory study
Source: BMC Health Serv Res. 2024 May 10;24:613. doi: 10.1186/s12913-024-11074-0 (PMC11084004; doi:10.1186/s12913-024-11074-0)
Supplement: Supplementary file 1 — Supplementary Material 1. [file 12913_2024_11074_MOESM1_ESM.docx]

**Key-informant and FGD Guide Questions**

**Part I: Consent form – key informant interview**

**Study Title**: **Inequity in The Utilization of Child HealthCare Services for under-five Children in Ethiopia:**

**A brief description:** This is a qualitative study that aims to understand and propose solution to reduce variation in child health care service utilization in Ethiopia. Data is collected through in-depth interviews and FGDs. The key stakeholders to be involved in this key informant interview includes but not limited to: Maternal and child health service providers (Health workers, Health Extension workers) in health centers and health posts, Arsi Zone health department head, MCH coordinator, Zuway Dugda Health office head, Woreda MCH coordinator and health centers head and HEW for each health posts. The study participants for FGDs are women who gave birth in the five years prior of the survey and their partners which will be selected with the assistance of health extension workers. Each FGD discussions and in-depth interviews will be tape recorded. Each FGD will take 1^1^/2-3 hours duration, while each in-depth interview is expected to last 45 minutes to 1 hour. Participants and their institutions identity will be protected; all data collected will only be accessed by the investigator only. Data will be stored in safe environment; all row data will be destroyed after analysis. The analysis and dissemination of result will be technically supported by the Addis Ababa University, College of Health Science. Ethical clearance will be attained from Addis Ababa University, College of Health Science. This study is part doctoral study by the investigator at Addis Ababa University, College of Health Science. If you agree, tape recorders will be used to record discussions. Participation in this study is voluntary and you can choose not to involve in any part of the study or you cannot answer some or any individual questions or all of the questions. However, we hope that you will participate fully in this assessment since your views are important. If you have any questions regarding the study or would like to be informed of the result after its completion you can contact the principal investigator.

**Investigator’s name and contact details:**

Hailu Fekadu Demessie

Email: hailufekadu18@yahoo.com

Phone: +251911717608

I have read the above information concerning this study [or have understood the verbal explanation] and I understand what will be required of me and what will happen to me if I take part in it.

My questions concerning this study have been answered by Hailu Fekadu Demessie.

I understand that at any time I may withdraw from this study without giving a reason and without being affected.

I, ________________________________________, agree to take part in this study.

I DO / DO NOT (please circle one) agree to quotations from my participation in the study to be included anonymously in reports about the study.

**Signed ……………………………. Date ……………………………………..**

I DO / DO NOT (please circle one) agree to get my interview recorded on tape for the purpose of transcription only.

**ANNEXX**

**Interview guide for Arsi Zone health office head, Woreda head, and health centers and Health Post Workers.**

**Intro:** Many thanks for agreeing to take part in this interview; your view on the subject matter will be invaluable. This interview will stay for about 45 minutes. If you agree, we will have a short tea break at the end of this discussion.

*NB: For KII facilitators/ modulators/*

- *Facilitate each discussion topics explicitly* deferred
- *Keep confidentiality!*

***Part I: Identification***

*Woreda----------------- kebele ----------------------------Date ---------------------------------------*

*Supervisor’s Name ------------------------------------------Name of KII facilitator---------------------------------- Name of Co- facilitator-----------------------*

***Part II: Basic Socio-demographic data of the KII participants***

| *Code of the participant* | *sex* | *Age* | *Educational status* | *Marital status* | *Religion* | *Residence* | *Monthly income* | *Occupation* |
| --- | --- | --- | --- | --- | --- | --- | --- | --- |
|  |  |  |  |  |  |  |  |  |
|  |  |  |  |  |  |  |  |  |
|  |  |  |  |  |  |  |  |  |

**Part III: Discussion topics on awareness of child healthcare services**

Start by introduction about the study protocol and its purpose

*Assigned duties and Responsibilities in line with Child health services*

*Then precede with Main discussion points.*

**Grand Tour Questions:**

In your view, what can be an example of the best attainable child health care service provision in Ethiopia today?

***Probe:*** *by types of services like, immunization and treatment of sick child*

*Search for examples in by residential areas: in urban and rural context, poor and rich*

**1. Idea**

**Theme 1: Awareness, Inequality versus inequity (fairness and right)**

• How do you describe the variation in child health service utilization and mortality rate difference among urban vs rural, different regions, and wealth groups of Ethiopia?

*Read out a line of description on the current inequality (mainly by wealth and place of residence) in Ethiopia, if the interviewee is not well aware of the facts*

• What types of disparities in health do you think are avoidable? What about child health service utilization and health disparities in Ethiopia?

• Follow up: *why? What do you think makes reduction/elimination of this disparity unavoidable?*

• Which one is very important for Ethiopia currently- increasing coverage or improving equity?

Given the limited budget for health which one of the two should be a priority for Ethiopia? *Why?*

• Do you believe the country has the resources to make child health care service utilization between rural and urban; the poorest and the richest; and the literate and illiterate to be similar? *Why*

**Theme 2: perception on the causes**

**Perceived and probable drivers of inequity**

• What do you think are the factors that caused these differences among Ethiopians in child health service utilization over a long period of time?

**Probe**: the effect of health systems, broader social determinants of health

• What kind of populations do you think don’t use child health services like *(medical treatment thought for childhood diarrhea, fever and cough, ITN utilization, exclusive breast feeding, immunization, Vit –A suplimentation)* probe poor vs rich, urban vs rural , literate vs illiterate male vs female and among different ethnic groups ?

• How does the health care system reduce or increase inequity in Ethiopia? Probe: Inequity by Wealth and region or urban rural differences.

**2. Institutional factors**

**Theme 3: organizational readiness, leadership and commitment**

• What guidelines/strategies do you have to reduce regional or urban rural variation in providing life-saving child health care services? For leaders

• What is the planning process at your office? How do you address the disparity in child health clinical services coverage Probe: (between rich/poor), (urban/rural) , literate vs illiterate male vs female and among different ethnic groups

***By Service types****: Immunization, skilled birth, treatment of sick child, ITN Utilization, Exclusive breast feeding, ORS therapy and vit-A supplementation etc.*

• How have you addressed the underserved poor and rural community in your strategic plan (HSTP)?

• Given the decentralized powers to districts, how does the districts, health centers and health posts operate in terms of reducing and monitoring disparities of service coverage?

**More details questions**

- In your opinion, what are the reasons/barriers that delay or prevent mothers to receive child health care service for their children?
- PROBE: what **cultural factors** are affecting a of women to obtain these services?[give focuses to immunization services, ORT,ITN utilization, vit A supplementation, exclusive breast feeding practice, health seeking behavior and medical treatment sought for diarrhea, fever and cough]
- PROBE: **on cultural factors** [culture, social value and norms of the community, religion, women’s autonomy]
- PROBE: what **financial factors** are affecting a of women to obtain these services?[give focuses to poor household, , low female autonomy, unemployment, education of mothers and fathers, health care cost]
- PROBE: what **educational barriers** are affecting a of women to obtain these services?[give focus on low female autonomy, unemployment, education of mothers and fathers, health care cost]
- PROBE: what **behavioral and psychological barriers**  are affecting a of women to obtain these services?[give focuses to, health seeking behavior and medical treatment sought for diarrhea, fever and cough, accessibility to mass media, perceived health status, availability of traditional healers, health promoting practices]
- PROBE: what **health system barriers** are affecting a women to obtain these services?[***inaccessibility to health facility, unavailability of the services, unaffordability of the service , quality of service, inadequate management system, and acceptability of the service , availability of companionate, respectful*** care]
- PROBE: what **ethnic barriers** are affecting a women to obtain these services?[give focuses to immunization services, ORT,ITN utilization, vit A supplementation, exclusive breast feeding practice, health seeking behavior and medical treatment sought for diarrhea, fever and cough]
- PROBE: on **ethnic minorities**
- PROBE: what **characteristics of birth** are affecting a women to obtain these services? [parity, place of birth, mode of delivery, sex of a child, child wanted and number of children]
- PROBE: what **distance/ regional barriers** are affecting a women to obtain these services?[transportation availability, distance to health facility, altitude of residence area and seasonal variation of child health service utilization]

**Theme 4: awareness on inverse equity and health systems**

• What targeted approaches/interventions do you use to reach people living in underserved woredas and kebeles? Remote areas,

• Please describe the process of budgeting and factors that determine budget distribution among districts and the amount that goes to the poorest? *For Zonal- level Interviews*

• What institutional set up do you have to support the distribution of child health drugs and commodities to remote health facilities?

• What is the role of fee retention, waive and exemption from fee in worsening or reducing this inequity?

• Follow up the same question for the health extension program and community mobilization techniques?

**Theme 5: the political drives and interest**

• What can be the political factors that you think play important role to improve awareness and response on health inequity among Ethiopians? particularly in your area?

• How is the government political policy and structures influence inequity in child health?

- How the current **conflict and political instability** is affect equity in child healthcare utilization?

**PROBE**: how and why it affect health system delivery?

**Theme 6: Attitude and values in appraising equity interventions**

• How do you think the poorest and underserved can be served equally as the others?

• What targeted programs do you have that are focused on the poorest/ Remote areas or regions?

• Health Equity Funds is a new model in Ethiopia that provides funds to the poorest section of communities to cover the cost of transportation, lodging and medical cost to reduce inequality fast. *What do you think about this initiative? How can your region/District or Ethiopia implement this?*

• Finally, what steps do you think are required for the country to introduce effective inequality reduction programs (poor vs rich) and regional disparities?

**Finally, summarize:**

- The socio cultural practices you understand on child health care services in your area
- The health promoting practices you understand on child health care services in your area
- The barriers you understand for child health care services utilization in this area.

We have finished

Many thanks for your time!!

**PART: II Focus Group Discussion**

**Study Title: *Inequity in The Utilization of Child Health Care Services for under-five Children in Ethiopia:***

Focus Group Discussion (FGD) guide for Women who gave birth within the last five year and their partners.

Date of FGD: Ethiopian (dd/mm/yyyy) ______ / ______ / ____________

*Woreda----------------- kebele ----------------------------*

*Name of FGD facilitator---------------------------------- Name of Note taker -----------------------*

**CONSENT FORM**

**[NB: Verbal consent should be taken from each FGD participants separately before starting discussion together]**

My name is____________. Currently I am working as qualitative data collector for the research topic: *inequity in the utilization of child health care Services among women of reproductive age group and their under-five children*.We are conducting this study to understand the utilization of child health services in this woreda. We are asking you to participate because you have given birth within the past five year and you are eligible to utilize the services. The information we learn from you and others in this study will help the District/zone to improve child health services in your district. It is not must for you to join, it is your choice.

**What is expected from the participants of the research study?**

If you agree to take part, you will be in a group discussion with up to 12 other women like you in this kebele. We will ask questions about your experiences in utilizing of child health services. No one else but women who take part in the discussion and the moderator and note-taker will be present during this discussion. The entire discussion will take approximately 90 minutes of your time.

With your permission, the group session will be audio-recorded. Then someone will write everything that was said from the audio file.  The information on the tapes will be kept safe and only the study team members will listen to them.  The recordings will be destroyed 3+ months after the spoken words have been written down.

**Privacy, anonymity and confidentiality:**

All research projects carry some risk that information about you may become known to people outside a study.  However, your name will not be attached to the personal information you give us or to any of the comments that you share during the group discussion. We may give you a number to refer to you during the discussion, and we ask that you not refer to other people by their true names.  What you say will be combined along with what others say, so your name will not be connected to what you have said.

Any comments made during the group discussion should not be discussed with people outside the group.  We cannot, however, guarantee that all participants will keep things confidential.

During the study, the information you share will only be used for this research purpose.

**Risks:** There will be no more than minimal risks related to your participation in this focus group discussion and study. There is a small risk that someone may overhear our conversation. We will try our best to keep your information private.

**Benefits:** You will not benefit directly from taking part in this study. You may be glad to hear about other women experiences similar to your own.

**Payment:** There is no payment for your participation. However, the discussion allows you to share your relevant experiences and opinions with us, and this helps us improve utilization of child health services in your community.

**Voluntary nature:** You do not have to help, it is your choice. You may choose not to answer any question that we ask.  You may leave the discussion at any time.

**Contacts:** You may contact Hailu Fekadu , the lead Investigator, at 0911717608 if you have questions or concern related to the study.

Do you have any questions?

Would you like to join the study?

____Yes ____No

**PAR I-INTRODUCTION**

***Welcome and introductions***

Thank you all very much for taking part in this focus group discussion. We have gone through informed consent with each of you. As a reminder, my name is [STATE NAME] and this is [STATE NAME OF NOTETAKER], who will be taking notes.

***Purpose of focus groups***

We are here to find out about your experience on Health seeking behavior and in utilizing of child health care services in your woreda. We have scheduled 90 minutes for our discussion today.

***Anonymity***

We will be audio recording the discussion but, when we write up the conversation, the transcript will not have any names. We will not be calling you by name; rather each of you has been given a number. You have all been through the informed consent process.

**Ground Rules**

To guide our discussion today, I will ask a series of questions. I encourage you all to speak up and share your thoughts and experiences. We want to hear many different opinions and everyone’s viewpoints are valuable. There is no right or wrong answers. We hope you can be honest even when your responses may not agree with the rest of the group.

***Keep information shared confidential***

In respect for each other, we ask that only one individual speak at a time in the group and that what is said during this conversation stays here. Please do not share what has been discussed outside the group.

***Questions?***

Does anyone have any questions? OK, I will start now.

***PART II: BASIC SOCIO-DEMOGRAPHIC DATA OF THE FGD PARTICIPANTS***

[Ask participants to introduce themselves *and fill the following table]*

| *Code of the participant* | *Age* | *Marital status* | *Religion* |  | *Residence* | *Educational status* | *Occupation* | *Av Income* | *Women’s current status* |
| --- | --- | --- | --- | --- | --- | --- | --- | --- | --- |
| *P_1_* |  |  |  |  |  |  |  |  |  |
| *P_2_* |  |  |  |  |  |  |  |  |  |
| *P_3_* |  |  |  |  |  |  |  |  |  |
| *P_4_* |  |  |  |  |  |  |  |  |  |
| *P_5_* |  |  |  |  |  |  |  |  |  |
| *P_6_* |  |  |  |  |  |  |  |  |  |
| *P_7_* |  |  |  |  |  |  |  |  |  |
| *P_8_* |  |  |  |  |  |  |  |  |  |
| *P_9_* |  |  |  |  |  |  |  |  |  |
| *P_10_* |  |  |  |  |  |  |  |  |  |
| *P_11_* |  |  |  |  |  |  |  |  |  |
| *P_12_* |  |  |  |  |  |  |  |  |  |

**QUESTIONS**

Thank you for telling us about yourselves. I will go ahead and start with the questions.

**PART II: Discussion topics on awareness of child healthcare services**

- 1. Do ***you know*** about child health services given to women and their children in health centers and health posts/community?
- PROBE: Ask them to list
- PROBE: Ask them to list all possible types of child health services
  1. Do you feel that health care service utilization is useful for mother and newborn?
     - PROBE: If yes, Ask them why?
     - PROBE: If no, Ask them why ?
     - PROBE: do you think should a woman will give birth at health facility? why?
     - PROBE: For how long do you think a women who gave birth in the facility should stayed in the facility before she and her baby is discharged? Why?
     - PROBE: What services she and her baby should be given before discharge? Why?
     - PROBE: when do you think a discharged women and her baby should visit facility? Why?
  2. Do you feel that child immunization services, is useful for children and family?
- PROBE: ORT,ITN utilization,
- PROBE: Vit A supplementation,
- PROBE: exclusive breast feeding practice,
- PROBE***: health seeking behavior and medical treatment sought for diarrhea, fever and cough***
  - - PROBE: If yes, Ask them why
    - PROBE: If no, Ask them why
    - PROBE: What do you think is a best time to start medical treatment sought for diarrhea, fever and cough after your child is sick? Why?
    - PROBE: Importance of ITN utilization
    - PROBE: child immunization time
    - PROBE: Exclusive breast feeding,
  1. Who do you think is a decision maker to obtain these child health services like immunization services, ORT,ITN utilization, vit A supplementation, exclusive breast feeding practice, health seeking behavior and medical treatment sought for diarrhea, fever and cough?

Why?

- PROBE: do you think women can independently decide to obtain these child health services? If no, why?
- PROBE: How involved are men and other family members in these child health decisions? Do you ever engage with these other people?

**More details questions**

- In your opinion, what are the **reasons/barriers that delay or** prevent mothers to receive child health care service for their children?
- PROBE: what **cultural barriers** are affecting a of women to obtain these services?[give focuses to immunization services, ORT,ITN utilization, vit A supplementation, exclusive breast feeding practice, health seeking behavior and medical treatment sought for diarrhea, fever and cough]
- PROBE: **on cultural barriers** [culture, social value and norms of the community, religion, women’s autonomy]
- PROBE: what **financial barriers** are affecting a of women to obtain these services?[give focuses to poor household, , low female autonomy, unemployment, education of mothers and fathers, health care cost]
- PROBE: what **educational barriers** are affecting a of women to obtain these services?[give focuses to low female autonomy, unemployment, education of mothers and fathers, health care cost]
- PROBE: what **behavioral and psychological barriers** are affecting a of women to obtain these services?[give focuses to, health seeking behavior and medical treatment sought for diarrhea, fever and cough, accessibility to mass media, perceived health status, availability of traditional healers, health promoting practices]
- PROBE: what **health system f barriers** are affecting a women to obtain these services?[inaccessibility to health facility, unavailability of the services, unaffordability of the service , quality of service, inadequate management system, and acceptability of the service , availability of companionate, respectful care]
- PROBE: what **ethnic f barriers** are affecting a women to obtain these services?[give focuses to immunization services, ORT,ITN utilization, vit A supplementation, exclusive breast feeding practice, health seeking behavior and medical treatment sought for diarrhea, fever and cough]
- PROBE: on **ethnic minorities**
- PROBE: what **characteristics of birth** are affecting a women to obtain these services? [parity, place of birth, mode of delivery, sex of a child, child wanted and number of children]
- PROBE: what **Distance/regional barriers** are affecting a women to obtain these services?[transportation availability, distance to health facility, altitude of residence area and seasonal variation of child health service utilization]
- PROBE: Ask them if there is obstacles/challenges related to information and accessibility to receive these services
  1. What ideas do you have or recommend to improve child health service utilization among mothers in this community?
  2. Anything else you would like to add?

**Thank you very much for your participation!**

**END TIME:** (HH:MM) **___ ___ : ___ ___**
